# Ambulatory systolic blood pressure and obesity are independently associated with left ventricular hypertrophic remodeling in children

**DOI:** 10.1186/s12968-017-0401-3

**Published:** 2017-11-09

**Authors:** Linyuan Jing, Christopher D. Nevius, Cassi M. Friday, Jonathan D. Suever, Arichanah Pulenthiran, Abba Mejia-Spiegeler, H. Lester Kirchner, William J. Cochran, Gregory J. Wehner, Aftab S. Chishti, Christopher M. Haggerty, Brandon K. Fornwalt

**Affiliations:** 1 Department of Imaging Science and Innovation, Geisinger, 100 North Academy Avenue, Danville, PA 17822-4400 USA; 2 Biomedical and Translational Informatics Institute, Geisinger, Danville, PA USA; 3 Pediatric Gastroenterology, Geisinger, Danville, PA USA; 4 Department of Biomedical Engineering, University of Kentucky, Lexington, KY USA; 5 Division of Nephrology, Hypertension and Renal Transplantation, University of Kentucky, Lexington, KY USA; 6 Department of Radiology, Geisinger, Danville, PA USA

**Keywords:** Pediatric obesity, Ventricular remodeling, Hypertension, Cardiovascular magnetic resonance, Ambulatory blood pressure monitoring

## Abstract

**Background:**

Children with obesity have hypertrophic cardiac remodeling. Hypertension is common in pediatric obesity, and may independently contribute to hypertrophy. We hypothesized that both the degree of obesity and ambulatory blood pressure (ABP) would independently associate with measures of hypertrophic cardiac remodeling in children.

**Methods:**

Children, aged 8–17 years, prospectively underwent cardiovascular magnetic resonance (CMR) and ABP monitoring. Left ventricular (LV) mass indexed to height^2.7^ (LVMI), myocardial thickness and end-diastolic volume were quantified from a 3D LV model reconstructed from cine balanced steady state free precession images. Categories of remodeling were determined based on cutoff values for LVMI and mass/volume. Principal component analysis was used to define a “hypertrophy score” to study the continuous relationship between concentric hypertrophy and ABP.

**Results:**

Seventy-two children were recruited, and 68 of those (37 healthy weight and 31 obese/overweight) completed both CMR and ABP monitoring. Obese/overweight children had increased LVMI (27 ± 4 vs 22 ± 3 g/m^2.7^, *p* < 0.001), myocardial thickness (5.6 ± 0.9 vs 4.9 ± 0.7 mm, *p* < 0.001), mass/volume (0.69 ± 0.1 vs 0.61 ± 0.06, *p* < 0.001), and hypertrophy score (1.1 ± 2.2 vs −0.96 ± 1.1, *p* < 0.001). Thirty-five percent of obese/overweight children had concentric hypertrophy. Ambulatory hypertension was observed in 26% of the obese/overweight children and none of the controls while masked hypertension was observed in 32% of the obese/overweight children and 16% of the controls. Univariate linear regression showed that BMI z-score, systolic BP (24 h, day and night), and systolic load correlated with LVMI, thickness, mass/volume and hypertrophy score, while 24 h and nighttime diastolic BP and load also correlated with thickness and mass/volume. Multivariate analysis showed body mass index z-score and systolic blood pressure were both independently associated with left ventricular mass index (β=0.54 [*p* < 0.001] and 0.22 [*p* = 0.03]), thickness (β=0.34 [*p* < 0.001] and 0.26 [*p* = 0.001]) and hypertrophy score (β=0.47 and 0.36, both *p* < 0.001).

**Conclusions:**

In children, both the degree of obesity and ambulatory blood pressures are independently associated with measures of cardiac hypertrophic remodeling, however the correlations were generally stronger for the degree of obesity. This suggests that interventions targeted at weight loss or obesity-associated co-morbidities including hypertension may be effective in reversing or preventing cardiac remodeling in obese children.

## Background

Childhood obesity affects 17% of children and adolescents (2–19 years) in the United States [1], and is associated with increased risk of cardiovascular disease and premature death [2, 3]. Although severe cardiovascular disease is rare in children, early signs of heart disease in obese/overweight children have been documented [4–7]. The most common findings include increased left ventricular (LV) mass and wall thickness. In addition, approximately 25% of obese/overweight children have concentric hypertrophy [7, 8]. These changes are worrisome as both increased LV mass and concentric hypertrophy have been related to increased cardiovascular risk and premature death in adults [9].

Mechanisms underlying hypertrophic cardiac remodeling in obese children are not well understood, at least in part because several obesity co-morbidities, particularly high blood pressure (BP), are known to independently cause LV hypertrophy [10]. High systolic BP, defined as systolic BP ≥95th percentile for height and sex [11], has a prevalence of up to 20% in obese/overweight children [12]. Elevated BP has also been independently associated with LV hypertrophy in childhood [13–15], and therefore increased risk for future adult cardiovascular disease [16].

Hypertension is commonly diagnosed with a repeated [11] clinic measurement (often referred to as casual hypertension), however, this approach provides poor characterization of actual BP [17]. Ambulatory blood pressure (ABP) monitoring provides a more accurate and comprehensive assessment of BP over a 24-h period. Previous studies have shown that compared to clinic BP, ABP has a stronger correlation with target organ damage (such as LV mass) in adults [18]. In addition, white-coat hypertension (elevated clinical BP but normal ambulatory BP (ABP) levels) and masked hypertension (normal clinic BP with elevated ABP levels) can only be diagnosed by ABP monitoring. Previous studies have reported a prevalence of 22–32% for white-coat and 7–32% for masked hypertension in children [19].

The relationship between ABP and LV hypertrophy in obese children is not well understood. A few studies using ABP monitoring have shown a positive correlation between systolic BP and increased LVMI [13–15, 20]. However, these studies either did not evaluate the independent effect of obesity [20], or were conducted on biased populations (children with casual hypertension [13], at risk for hypertension [14], or with other complications [15]). To our knowledge, no study has comprehensively investigated the relationship between obesity, ABP and measures of cardiac remodeling in otherwise healthy children.

In addition, all previous studies used echocardiography to assess LV mass and/or thickness. Transthoracic ehocardiography suffers from limited acoustic windows and angle dependency. Cardiovascular magnetic resonance (CMR) imaging overcomes the above limitations, and is therefore the ideal tool for definitively assessing cardiac geometry and remodeling. The objective of this study was to comprehensively evaluate the relationship between obesity, ABP measurements and cardiac remodeling in uncomplicated, asymptomatic children without and with obesity. We hypothesized that ABP measurements and obesity would both independently correlate with CMR derived measures of cardiac remodeling (mass, thickness, hypertrophy).

## Methods

### Study population

Children ages 8–17 years were prospectively recruited from the University of Kentucky (the High BMI Diagnostic Clinic, and the Center for Clinical and Translational Science volunteer database) and Geisinger Medical Center. Body mass index (BMI) percentiles for age and gender based on the Centers for Disease Control growth charts [21] were used to group the children into different weight categories: obese/overweight (BMI ≥85th percentile) and healthy weight (BMI 5th–85th percentile). Children were excluded if they had 1) diabetes, 2) diagnosed hypertension or history of taking medications that could alter BP, 3) history of heart disease, or 4) contraindications for CMR (including a waist circumference > 125 cm due to the circumference limitation of the scanner bore). A subset (one third) of the subjects were included in a previous study on LV remodeling and cardiac strain [7].

### Clinical assessment

Clinical assessment took place at the time of the CMR scan. Height and weight were measured twice using a digital scale and the average values were used to determine age and sex specific BMI (weight/height^2^ in kg/m^2^) percentiles. Resting BP was measured manually by auscultation using an appropriately sized cuff after 10 min of rest. Three measurements were taken, and the average of the last two was reported as the clinic BP. The clinic BP was classified into normal, pre-hypertensive or hypertensive based on established reference values for age, height and sex. All children had a normal 12-lead electrocardiogram.

### CMR imaging

A CMR study was performed on all subjects on a 3 T (Trio, Siemens Healthineers, Erlangen, Germany) using 6-element chest and 24-element spine coils. Standard elecrocardiogram-gated balanced steady-state free-precession (bSSFP) images were acquired during 10–15 s breath-holds to assess cardiac geometry and remodeling. Two-chamber, four-chamber and a stack of short-axis bSSFP images spanning both ventricles were acquired. 7–11 short-axis images were acquired depending on the size of the heart. Acquisition parameters included 3.16−3.37 ms repetition time, 1.3−1.5 ms echo time, [292−400] x [340−400] mm^2^ field of view, [208−256] × 256 image matrix, 50° flip angle, 16.4−49.9 ms temporal resolution, 8 mm slice thickness, and 0−3.7 mm slice gap.

### Cardiac remodeling

**Fig. 1:**
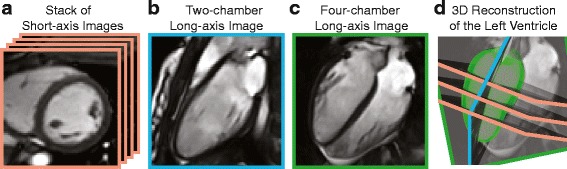
Three-dimensional left ventricular (LV) endocardial and epicardial surfaces (**d**) were reconstructed using a stack of short-axis bSSFP images (**a**), a two-chamber view (**b**), and a four-chamber view (**c**)

To assess cardiac geometry and remodeling, both LV and right ventricular (RV) endocardial boundaries were manually delineated on end-diastolic and end-systolic frames of the bSSFP images. LV epicardial boundaries were defined on an end-diastolic frame to quantify LV mass and wall thickness. LV end-diastolic (EDV) and end-systolic (ESV) volumes were computed from reconstructed 3D endocardial surfaces (Figure 1) using a custom algorithm written in MATLAB (The Mathworks, Natick, Massachusetts, USA) as previously described [7]. A 3D epicardial surface was reconstructed to quantify LV myocardial mass assuming a myocardial density of 1.05 g/mL. To account for somatic growth, LV mass was indexed to height^2.7^ (LVMI), which has been shown to best predict cardiac risk compared to other methods of normalization [22]. LV myocardial wall thickness was computed as the equipotential distance between the epicardial and endocardial surfaces for over 2000 points on the 3D surfaces [23]. The average of all distances was reported as the mean thickness. RV EDV and ESV were calculated using Simpson’s rule using contours from all short-axis images. RV myocardial mass (indexed to height^2.7^, RVMI) was calculated from the end-diastolic frame assuming a myocardial density of 1.05 g/mL. LV and RV ejection fraction were also derived ((EDV-ESV)/EDVx100%).

**Fig. 2:**
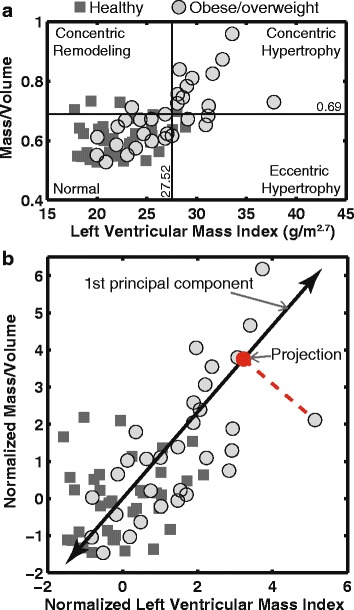
Principal component analysis (PCA) was used to represent left ventricular (LV) concentric hypertrophy. LV remodeling types were determined using previously defined cutoff values of LV mass index (LVMI) and mass/volume ratio (**a**). LVMI and mass/volume ratio were normalized by subtracting the mean and divided by the standard deviation derived from the healthy controls, and then hypertrophy scores were defined as the projections (red dot) along the 1st principal component direction using PCA (**b**)

To study the relationship between hypertrophic cardiac remodeling and BP measurements, cutoff values of LVMI (27.52 g/m^2.7^) and mass/volume ratio (0.69), defined from a previous study [7], were used to classify all subjects into one of the four LV remodeling types: normal geometry, concentric remodeling, eccentric hypertrophy and concentric hypertrophy (Figure 2A). Furthermore, to represent the presence of concentric hypertrophy with a continuous variable, principal component analysis using LVMI and mass/volume was used to derive a hypertrophy score. LVMI and mass/volume were normalized by the mean and standard deviation (SD) of the healthy controls, and hypertrophy score was defined as the projection onto the first principal component (Figure 2B). A lower hypertrophy score corresponds to a more normal geometry, whereas a higher score corresponds to more concentric hypertrophy.

### Ambulatory blood pressure monitoring

Following the CMR scan, ABP monitoring was conducted using a *SunTech Oscar 2 24-h ABP device and Orbit BP cuff* (SunTech Medical, Inc. Morrisville, North Carolina, USA)*.* An appropriate cuff based on the subject’s arm circumference was chosen from four available sizes, and placed tightly on the subject’s non-dominant arm. The device would inflate shortly after activation and take two readings within the first 5 min, which a member of the study team used to confirm correct functionality. These initial readings were not included in subsequent analysis. The device was programmed to take a reading every 20–30 min between 7:00 am and 10:00 pm, and every 30–45 min from 10:00 pm to 7:00 am. Daytime and nighttime for each subject were determined by self-reported sleep and wake times from diaries. Subjects were advised to follow ordinary daily activities but avoid vigorous exercise and to relax the arm during the inflation and deflation of the cuff. Measurements were automatically repeated if the device failed to take a reading.

After 24 h, the device and cuff were removed. Data were downloaded using the manufacturer’s software *AccuWin Pro*, and analyzed according to the American Heart Association (AHA) statement [19]. Subjects (*n* = 4) with fewer than 14 daytime readings or fewer than 7 nighttime readings were excluded from analysis. Height- and sex- specific normative reference values from a European cohort provided by the German Working Group on Pediatric Hypertension were used [24]. BP load was defined as the percentage of measurements that were above the 95th percentile for height and sex. Mean systolic BP (SBP) and diastolic BP (DBP), and systolic and diastolic loads were computed for the entire 24-h period, day time and night time. In addition, systolic and diastolic dipping were calculated as the percent drop in mean nighttime BP relative to mean daytime BP ((BP_day_-BP_night_)/BP_day_x100%). A dipping of at least 10% was considered normal [19]. BP staging was determined based on classification criteria from the American Heart Association statement [19] using both clinic BP and ABP measurements and summarized as follows:

1. Normal BP: clinic BP <90th percentile, ABP <95th percentile and BP load <25%;
2. Masked hypertension: clinic BP <95th percentile, ABP >95th percentile and BP load ≥25%;
3. Pre-hypertension: clinic BP ≥90th percentile, ABP <95th percentile and BP load ≥25%;
4. Ambulatory or sustained hypertension: both clinic BP and ABP >95th percentile and BP load ≥25%.

### Statistics

Continuous variables were reported as mean ± SD. The two-sample student’s t-test was used to compare differences between the obese/overweight and healthy groups. Categorical variables were compared between groups using either Pearson’s Chi-Square or Fisher’s exact tests. Linear regression, with adjustment for age, was used to estimate the differences in measures of cardiac remodeling and ABP parameters between groups. Correlations between BMI z-score, BP parameters and measures of cardiac remodeling were estimated using Pearson’s correlation coefficient. Stepwise linear regression was performed to determine independent predictors for measures of cardiac remodeling. Akaike information criterion (AIC) was used for model selection, i.e. the model with the smallest AIC was selected as the final model by the stepwise regression. Statistical significance level was set to *p* < 0.05. All statistical analyses were performed in R [25] (Version 3.3.1).

## Results

### Demographics and clinical assessment

Seventy-two children were enrolled in the study. Of those, 4 did not complete ABP monitoring and were excluded from data analysis. A total of 68 subjects, including 31 obese/overweight (median age: 12.5 years, interquartile range: 11.3–14.3 years, 43% female) and 37 healthy weight (median age: 13.3 years, interquartile range: 12.1–15.6 years, 55% female) children, completed both CMR and ABP monitoring and were included in subsequent analysis. Table 1 summarizes the demographics and clinical assessment of the study population. Age, sex and height were comparable between the two groups.

**Table 1:** Demographics and clinical parameters (mean ± SD, and median [interquartile range]) of the study population

	Obese/Overweight *n* = 31	Healthy *n* = 37	*p* ^*^
Age (years)	12.8 ± 2.5 12.5 [11.3, 14.3]	13.4 ± 2.6 13.3 [12.1, 15.6]	0.31
Sex (M/F)	14/17	21/16	0.47
Weight (kg)	75 ± 21 74 [56, 92]	47 ± 13 49 [37, 56]	*<0.001*
Height (cm)	158 ± 13 157 [149, 165]	156 ± 14 158 [147, 166]	0.56
Body Mass Index (kg/m^2^)	29 ± 6 29 [25, 33]	19 ± 3 19 [18, 21]	*<0.001*
Body Mass Index Percentile	96 ± 4 98 [95, 99]	47 ± 26 52 [27, 66]	
Body Mass Index z-score	2.0 ± 0.5 2.2 [1.7, 2.3]	−0.2 ± 0.9 0.1 [−0.6, 0.4]	
Heart rate (beats/min)	72 ± 9	70 ± 9	0.32
Systolic blood pressure (mmHg)	117 ± 11	111 ± 8	*0.001*
Diastolic blood pressure (mmHg)	75 ± 6	72 ± 5	0.07
Mean arterial pressure (mmHg)	89 ± 7	85 ± 6	*0.005*

*p values for systolic, diastolic and mean blood pressures are adjusted for age

### Cardiac remodeling

**Fig. 3:**
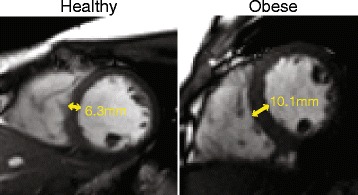
A representative example of LV remodeling (wall thickening) from an obese male with concentric hypertrophy compared to an age-matched healthy male

LVMI (27 ± 4 vs 22 ± 3 g/m^2.7^, *p* < 0.001), mean myocardial thickness (5.6 ± 0.9 vs 4.9 ± 0.7 mm, *p* < 0.001) and mass/volume ratio (0.69 ± 0.1 vs 0.61 ± 0.06, *p* < 0.001) were significantly larger in obese/overweight children compared to healthy controls (Table 2). A representative example of LV remodeling is shown in Fig. 3. In addition, obese/overweight children had larger RVMI (7.9 ± 1.2 vs 6.5 ± 1.0 g/m^2.7^, *p* < 0.001). LV and RV EDV and ESV were comparable between the groups. There were no significant differences in LV or RV ejection fractions (Table 2).

**Table 2:** Cardiac geometry, ejection fraction and remodeling, mean ± SD or N(%)

	Obese/Overweight *n* = 31	Healthy *n* = 37	*p, age adjusted*
LV geometry and function			
LV mass index (g/m^2.7^)	27 ± 4	22 ± 3	*<0.001*
LV end-diastolic volume (mL)	137 ± 29	125 ± 36	0.07
LV end-systolic volume (mL)	51 ± 14	48 ± 15	0.25
LV mass/volume ratio	0.69 ± 0.10	0.61 ± 0.06	*<0.001*
LV mean thickness (mm)	5.6 ± 0.9	4.9 ± 0.7	*<0.001*
LV ejection fraction (%)	63 ± 4	62 ± 4	0.44
LV remodeling			
LV hypertrophy score	1.1 ± 2.2	−0.96 ± 1.1	*<0.001*
LV remodeling types*			*<0.001*
Normal geometry	15 (49)	31 (84)	
Concentric remodeling	1 (3)	3 (8)	
Eccentric hypertrophy	4 (13)	2 (5)	
Concentric hypertrophy	11 (35)	1 (3)	
RV geometry and function			
RV mass index (g/m^2.7^)	7.9 ± 1.2	6.5 ± 1.0	*<0.001*
RV end-diastolic volume (mL)	151 ± 37	141 ± 42	0.20
RV end-systolic volume (mL)	59 ± 18	55 ± 20	0.37
RV ejection fraction (%)	61 ± 6	61 ± 4	0.94

*LV* left ventricular, *RV* right ventricular

*p value for LV remodeling types is not adjusted for age

More than half of the obese/overweight children had some form of LV remodeling: 11 (35%) had concentric hypertrophy, 1 (3%) had concentric remodeling, 4 (13%) had eccentric hypertrophy, and the remaining 15 (49%) had normal geometry. The hypertrophy score was also higher in the obese/overweight children compared to the healthy weight group (1.1 ± 2.2 vs −0.96 ± 1.1, *p* < 0.001, Table 2).

### Blood pressure measurements

Compared to healthy controls, obese/overweight children had elevated clinic SBP (117 ± 11 vs 111 ± 8 mmHg, *p* = 0.001) and mean arterial pressure (89 ± 7 vs 85 ± 6 mmHg, *p* = 0.005) (Table 1). Results of the ABP measurements are summarized in Table 3. SBP and systolic load in obese/overweight children was elevated for all time periods compared to healthy controls. Specifically, 24 h SBP was elevated by 8% (*p* = 0.004), while systolic load almost doubled that of the healthy controls (*p* = 0.001). In addition, obese/overweight children had slightly higher nighttime DBP (59 ± 9 vs 56 ± 6 mmHg, *p* = 0.046) and 24 h diastolic load (19 ± 17% vs 12 ± 11%, *p* = 0.04), while 24 h and daytime DBP and daytime and nighttime diastolic load were comparable to healthy controls. Systolic dipping (10 ± 6% vs 13 ± 7%, *p* = 0.09) and diastolic dipping (15 ± 9% vs 19 ± 8%, *p* = 0.07) trended lower in obese/overweight children.

**Table 3:** Ambulatory blood pressure monitoring measurements (mean ± SD)

	Obese/Overweight *n* = 31	Healthy *n* = 37	*p, age adjusted*
24-h			
24 h SBP (mmHg)	126 ± 15	117 ± 11	*0.004*
24 h DBP (mmHg)	66 ± 8	64 ± 6	0.19
24 h systolic load (%)	41 ± 28	22 ± 18	*0.001*
24 h diastolic load (%)	19 ± 17	12 ± 11	*0.04*
Daytime			
Day SBP (mmHg)	130 ± 15	123 ± 11	*0.01*
Day DBP (mmHg)	70 ± 8	69 ± 7	0.58
Day systolic load (%)	41 ± 28	23 ± 17	*0.001*
Day diastolic load (%)	17 ± 16	13 ± 14	0.21
Nighttime			
Night SBP (mmHg)	117 ± 17	107 ± 13	*0.003*
Night DBP (mmHg)	59 ± 9	56 ± 6	*0.046*
Night systolic load (%)	41 ± 31	20 ± 26	*0.003*
Night diastolic load (%)	22 ± 24	13 ± 15	0.06
Nocturnal Dipping			
Systolic dipping (%)	10 ± 6	13 ± 7	0.09
Diastolic dipping (%)	15 ± 9	19 ± 8	0.07

*SBP* systolic blood pressure, *DBP* diastolic blood pressure

### Blood pressure classifications

BP classification was significantly different between obese/overweight and healthy weight children (*p* < 0.001, Table 4). Eight out of the 31 (26%) obese/overweight children had ambulatory hypertension compared to none in the healthy weight group. The prevalence of masked hypertension was 32% in obese/overweight children, compared to 16% in healthy controls. The prevalence of pre-hypertension was low and comparable between the groups (6% in obese/overweight vs 5% in healthy weight). Only 36% of obese/overweight children had normal BP.

**Table 4:** Blood pressure classification, N(%)

Classification	Obese/Overweight *n* = 31	Healthy *n* = 37	Total *n* = 68
Normal	11 (36)	29 (79)	40 (59)
Masked hypertension	10 (32)	6 (16)	16 (23)
Pre-hypertension	2 (6)	2 (5)	4 (6)
Ambulatory hypertension	8 (26)	0 (0)	8 (12)

*p* < 0.001 between obese/overweight and healthy weight children

### Correlations between blood pressure, obesity and cardiac remodeling

Results of the univariate linear regression between ABP measurements, BMI z-score and measures of cardiac remodeling are reported in Table 5 where only significant correlations are shown. BMI z-score moderately correlated to all measures of cardiac remodeling (LVMI: *r* = 0.62; mean thickness: *r* = 0.49; mass/volume: *r* = 0.43; hypertrophy score: *r* = 0.58; all *p* < 0.001).

**Table 5:** Linear correlations between BMI z-score, ABP measurements and measures of left ventricular remodeling

	LVMI		LV Thickness		LV Mass/Volume		LV Hypertrophy score	
	*r*	*p*	*r*	*p*	*r*	*p*	*r*	*p*
BMI z-score	0.62	<0.001	0.49	<0.001	0.43	<0.001	0.58	<0.001
Systolic								
24 h SBP	0.36	0.003	0.60	<0.001	0.44	<0.001	0.44	<0.001
Day SBP	0.39	0.001	0.57	<0.001	0.41	<0.001	0.45	<0.001
Night SBP	0.34	0.005	0.57	<0.001	0.46	<0.001	0.43	<0.001
24 h systolic load	0.33	0.006	0.43	<0.001	0.36	0.003	0.37	0.002
Day systolic load	0.34	0.005	0.37	0.002	0.32	0.007	0.36	0.003
Night systolic load	0.31	0.01	0.44	<0.001	0.39	0.001	0.36	0.003
Systolic dipping					−0.26	0.04		
Diastolic								
24 h DBP					0.27	0.03		
Night DBP			0.24	0.046	0.31	0.01		
24 h diastolic load			0.26	0.03	0.31	0.009		
Night diastolic load			0.32	0.007	0.32	0.009		

*ABP* ambulatory blood pressure, *BMI* body mass index, *SBP* systolic blood pressure, *DBP* diastolic blood pressure, *LVMI* left ventricular mass index

LVMI and hypertrophy score correlated with all measures of SBP and systolic load, but most strongly with 24 h SBP (LVMI: *r* = 0.36, *p* = 0.003; hypertrophy score: *r* = 0.44, *p* < 0.001). Mean thickness and mass/volume correlated to all BP and BP load measurements except for daytime DBP. The strongest correlations were also with 24 h SBP (thickness: *r* = 0.60, *p* < 0.001; mass/volume: *r* = 0.44, *p* < 0.001). There was also a weak negative correlation between mass/volume and systolic dipping (*r* = −0.26, *p* = 0.04).

### Multivariate linear regression

**Fig. 4:**
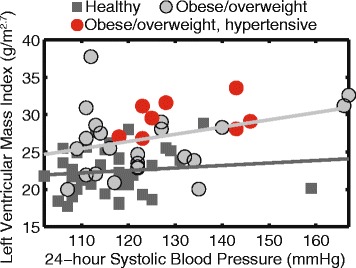
24 h systolic blood pressure is associated with left ventricular mass index (LVMI), however, obesity independently contributes to increased LVMI

After adjusting for sex and height, BMI z-score and 24 h SBP were independent predictors of LVMI (β=0.54 and 0.22) and hypertrophy score (β=0.47 and 0.36), while nighttime SBP and BMI z-score were independent predictors of thickness (β=0.26 and 0.34) and mass/volume ratio (β=0.35 and 0.31) (Table 6). Moreover, Fig. 4 shows that two distinct regression models describe the relationship between 24 h SBP and LVMI for obese/overweight children and healthy weight children, indicating independent contributions of 24 h SBP and obesity to increased LVMI. BMI z-score generally had stronger relationships (higher *β* coefficients) with the measures of LV remodeling compared to the ABP derived metrics.

**Table 6:** Multivariate linear regression (height and sex adjusted)

	LVMI		LV Thickness		LV Mass/Volume		LV Hypertrophy Score	
	*β (SE)*	*p*	*β (SE)*	*p*	*β (SE)*	*p*	*β (SE)*	*p*
BMI z-score	0.54 (0.10)	<0.001	0.34 (0.07)	<0.001	0.31 (0.10)	0.004	0.47 (0.10)	<0.001
24 h SBP	0.22 (0.10)	0.03					0.36 (0.10)	<0.001
Night SBP			0.26 (0.08)	0.001	0.35 (0.11)	0.002		

*β: normalized coefficient, SE standard error, BMI* body mass index, *SBP* systolic blood pressure, *LVMI* left ventricular mass index

## Discussion

In the current study, we comprehensively investigated the relationship between obesity, ambulatory blood pressure and measures of cardiac remodeling using CMR in 68 asymptomatic children. Major findings include: 1) obese/overweight children have LV and RV remodeling, as evidenced by increased LVMI, LV wall thickness, LV mass/volume and RVMI; 35% of obese/overweight children have concentric LV hypertrophy; 2) ambulatory SBP, DBP and BP loads are elevated in obese/overweight children; 26% of obese/overweight children have ambulatory hypertension and 32% have masked hypertension; 3) BMI z-score, systolic BP and BP load correlate with all measures of LV remodeling (LVMI, thickness, mass/volume, hypertrophy score); 4) BMI z-score and 24 h SBP independently associate with LVMI and the extent of LV concentric hypertrophy.

### Obesity-related hypertension using ambulatory blood pressure monitoring

Hypertension is a common comorbidity in childhood obesity [26]. In multiple cross-sectional and longitudinal studies conducted in children, BMI has been shown to have a strong effect on increases in BP that is greater than all other considered factors [27–29]. Considering the well-known effect of hypertension on cardiovascular morbidity in adults [16], accurate assessment and diagnosis of obesity-related hypertension is critical to appropriately risk stratify these children and consider targeted treatment. Clinic BP is a commonly used tool for screening subjects with hypertension. However, this single measurement of BP may not reflect physiological variations in BP, leading to an inaccurate or missed diagnosis. ABP monitoring provides a comprehensive evaluation of the BP profile and is therefore superior to clinic BP in detecting hypertension in obese children.

Based on American Heart Association criteria [19], 26% of obese/overweight children had ambulatory/sustained hypertension. This prevalence is lower than the 50–60% range reported by previous studies [30–32], likely because we only enrolled uncomplicated and asymptomatic subjects without a clinical diagnosis of hypertension. These criteria may similarly explain why we did not detect any subjects with white coat hypertension, although a prevalence of 30–50% has been reported previously [33, 34]. Additionally, masked hypertension was detected in 32% of obese/overweight and 16% of healthy weight children. The true prevalence of masked hypertension is not well established in the literature, ranging from 7% in the general population [31] to 26% in subjects at risk for hypertension [32]. The modest discrepancies may lie in the variations in sample size and selection bias of the study population, and a larger population may be needed to determine the true prevalence of masked hypertension in children. Identification of masked hypertension is important since these children may have similar cardiovascular risk as those with sustained hypertension [35, 36]. ABP monitoring is therefore an essential tool for risk stratification of hypertensive children.

### Obesity, blood pressure and cardiac remodeling

Cardiac remodeling, estimated by increased LVMI, wall thickness and the presence of LV hypertrophy defined as LVMI > 51 g/m^2.7^ using echocardiography [37], has been widely used as a surrogate for target organ damage in the pediatric population [13, 15, 38, 39]. Children with increased LVMI and LV hypertrophy may be at increased risk of cardiovascular disease and premature death as adults [16]. Therefore, identifying mechanisms underlying cardiac remodeling is essential for targeted treatment.

Obesity and hypertension have both been related to cardiac remodeling in children. Although obesity is known to impact LV geometry independent of its associated risk factors such as insulin resistance and inflammatory biomarkers [8, 19, 38, 40], reports on the role of hypertension in cardiac remodeling are controversial. Increased LVMI is often linked to hypertension assuming the ventricular wall thickens to compensate for increased afterload. Although most studies have shown that after controlling for BMI z-score, ambulatory systolic BP and/or systolic load correlated with LVMI and/or relative wall thickness [13–15, 32], a few studies found no [38] or weak [34] associations between elevated BP and increased LVMI or LV hypertrophy. An observational study by Brady et al. [38] further showed that between two clinic visits 12 months apart, despite increased LVMI and prevalence of LV hypertrophy at the second visit, change in BP was minimal; and that adiposity remained the only factor independently associated with increased LVMI after adjusting for multiple biomarkers. Similarly, in obese adults who lost weight through bariatric surgery, decreases in LVMI and relative wall thickness were not associated with a reduction in BP [41]. This evidence suggests that LV remodeling in obese children is mediated through both BP dependent and independent pathways.

In the current study, to comprehensively evaluate the contribution of obesity and blood pressure to cardiac remodeling in obese children, we used CMR with 3D surface reconstructions to assess LV geometry and remodeling, and defined a continuous variable (the hypertrophy score) to represent the presence of LV hypertrophy. CMR is superior to 2D transthoracic echocardiography for quantification of cardiac geometry and remodeling due to better image quality and inter-observer and inter-test reproducibility [42], while echocardiography suffers from limited acoustic windows and angle dependency. Note that the reported LVMI in the current study is smaller than the previously defined LV hypertrophy threshold value of 51 g/m^2.7^, probably due to multiple reasons. First, the 51 g/m^2.7^ threshold was defined using echocardiography, and is not directly applicable to CMR since discrepancies between CMR and echocardiography have been reported [43]. Second, our study was conducted in children, while the 51 g/m^2.7^ threshold was defined in adults. Therefore, we used threshold values of LVMI and mass/volume ratio derived from a previous CMR study in healthy children to categorize the different remodeling types [7].

Consistent with most previous studies, we found that both BMI z-score and systolic BP (24 h or nighttime) are independently associated with LVMI, thickness, mass/volume and hypertrophy score. Moreover, compared to systolic BP, BMI z-score may be more strongly associated with measures of cardiac remodeling, especially for LVMI. When plotting LVMI against 24 h systolic BP, LVMI for obese/overweight children fit on a different line from healthy weight children. This finding suggests that although LV remodeling is affected by elevated BP to some extent, it may not be the most important contributor. Thus, while antihypertensive treatment may be warranted in children with hypertension, additional interventions targeted at weight loss or pathways involved in other obesity co-morbidities may be necessary to effectively reverse or prevent cardiac remodeling and future cardiovascular risk.

### Limitations

In this cross-sectional study, contributions of obesity and ABP to changes in measures of cardiac remodeling could not be investigated. Future studies with longitudinal follow-up are required to address this issue. In addition, most subjects in the current study were white. As racial differences have been shown to affect ABP in children [44], results observed in the current study may not hold true for a more generalized pediatric population. However, a previous study has shown that race/ethnicity does not affect the relationship between BP and LVMI [14]. Since gender also impacts the relationship between obesity, BP and cardiac remodeling [45], we included gender as a dependent variable in the multivariate model. However, we did not find significant correlations between gender and measures of cardiac remodeling in the current study.

Multiple statistical tests were performed in the current study, however, we chose not to adjust for multiple testing and to leave it to the reader to interpret the statistical results in the presence of multiple testing. The associations between ABP and cardiac outcomes were investigated using linear models, it is possible that the associations were nonlinear. However, an assessment of the linearity assumption for the continuous variables was performed and all were found to be linear.

## Conclusions

In children, both the degree of obesity and elevated ambulatory blood pressure are independently associated with increased LVMI and wall thickness as well as the presence of concentric hypertrophy. This suggests that interventions targeted at weight loss or pathways involved in obesity-associated co-morbidities such as hypertension may be effective in reversing or preventing cardiac remodeling and future cardiovascular risk.

## Abbreviations

ABP: Ambulatory blood pressure
BMI: Body mass index
BP: Blood pressure
bSSFP: Balanced steady-state free-precession
CMR: Cardiovasular magnetic resonance
DBP: Diastolic blood pressure
EDV: End-diastolic volume
ESV: End-systolic volume
LV: Left ventricle/left ventricular
LVMI: Left ventricular mass index
RV: Right ventricle/right ventricular
RVMI: Right ventricular mass index
SBP: Systolic blood pressure
SD: Standard deviation
